# The Impact of Diabetes and Metabolic Syndrome Burden on Pain, Neuropathy Severity and Fiber Type

**DOI:** 10.1002/acn3.70072

**Published:** 2025-05-19

**Authors:** Long Davalos, Brian C. Callaghan, Lavanya Muthukumar, Simone Thomas, Evan L. Reynolds, A. Gordon Smith, J. Robinson Singleton, Ahmet Höke, Senda Ajroud‐Driss, Mazen M. Dimachkie, Stefanie Geisler, David M. Simpson, PNRR Study Group, Amro M. Stino

**Affiliations:** ^1^ Department of Neurology Kansas University Medical Center Kansas City Kansas USA; ^2^ Department of Neurology University of Michigan School of Medicine Ann Arbor Michigan USA; ^3^ Department of Neurology Johns Hopkins University School of Medicine Baltimore Maryland USA; ^4^ Department of Neurology Virginia Commonwealth University Richmond Virginia USA; ^5^ Department of Neurology University of Utah School of Medicine Salt Lake City Utah USA; ^6^ Department of Neurology Northwestern University Feinberg School of Medicine Chicago Illinois USA; ^7^ Department of Neurology Washington University in St. Louis School of Medicine St. Louis Missouri USA; ^8^ Department of Neurology Icahn School of Medicine at Mount Sinai Medical Center New York City New York USA

**Keywords:** cryptogenic sensory polyneuropathy, diabetic neuropathy, idiopathic neuropathy, metabolic syndrome, metabolic syndrome burden

## Abstract

**Objective:**

Determine the association between diabetes and metabolic syndrome (MetS) burden (number of MetS criteria fulfilled) and pain, neuropathy severity, and fiber type involvement in individuals with established polyneuropathy.

**Methods:**

The Peripheral Neuropathy Research Registry was queried for individuals with type 1 and type 2 diabetes (DPN) and non‐diabetic peripheral neuropathy (cryptogenic sensory polyneuropathy and prediabetes) using cross‐sectional observational data. Associations between diabetes or MetS burden and pain presence (yes/no), neuropathy severity (Total Neuropathy Score reduced), and fiber type involvement (pinprick, vibration, and proprioception examination—small, large, mixed) using logistic, linear, and multinomial regression models were determined.

**Results:**

A total of 1112 participants were included (265 DPN, 847 non‐diabetic peripheral neuropathy [NDPN]). Compared to NDPN, DPN participants were more likely to have pain, higher neuropathy severity, and mixed fiber involvement. In adjusted models, diabetes was associated with pain (odds ratio [OR] 1.85, CI: 1.15–3.03) and severity (point estimate [PE] 0.84, CI: 0.27–1.42), but not fiber type involvement. As the MetS burden increased, pain, neuropathy severity, and mixed fiber type involvement increased (*p* < 0.05 for trend). In adjusted models, MetS burden was associated with pain (OR 1.23, CI: 1.06–1.41) but not severity or fiber type involvement.

**Interpretation:**

Participants with DPN were more likely to have pain, greater neuropathy severity, and possibly more mixed fiber involvement than those with NDPN. Similarly, increasing MetS burden also led to more painful neuropathy and possibly more severe neuropathy with more mixed fiber involvement.

## 1 Introduction

Diabetes mellitus (DM) is the most common cause of peripheral neuropathy (PN), accounting for nearly half of all cases [1]. Further, diabetic peripheral neuropathy (DPN) affects nearly 50% of individuals with DM [2, 3, 4]. DPN is the fifth most common cause of neurological disability worldwide [5] and is associated with significant health care costs. The global health expenditure in 2021 due to DPN was $966 billion [6], while annually in the United States, DPN costs $10 billion [7]. Metabolic syndrome (MetS) is now recognized as a central contributor to the pathogenesis of DPN, especially in individuals with type 2 DM (T2DM). MetS is also a risk factor for otherwise cryptogenic sensory polyneuropathy (CSPN), which constitutes nearly 25%–30% of all polyneuropathy cases [8, 9].

International population‐based studies have firmly identified MetS as a risk factor for neuropathy [10, 11, 12, 13, 14, 15, 16]. MetS increases the likelihood of neuropathy, independent of glycemic status, but also accelerates the rate of DPN progression in individuals with established DM [17]. Obesity, particularly central obesity, is the second most important metabolic risk factor after hyperglycemia [10, 11, 18]. Importantly, a higher MetS burden (presence of more MetS components) increases DPN likelihood, accelerates DPN progression, and shortens the time to symptom onset [10, 11, 19, 20].

While the relationship between both diabetes and MetS burden with neuropathy prevalence is well established, the association between diabetes and MetS burden with pain, neuropathy severity, and fiber type involvement among individuals with polyneuropathy has not been firmly established.

In this study, we (1) compared pain prevalence, neuropathy severity, and fiber type involvement in individuals with DPN and non‐diabetic peripheral neuropathy (NDPN), (2) determined the trend between increasing MetS burden and these outcomes, and (3) evaluated associations between diabetes and MetS burden and these three neuropathy outcomes, using cross‐sectional data from the Peripheral Neuropathy Research Registry (PNRR).

## 2 Methods

### 2.1 Population and Study Design

PNRR is a multicenter database and biorepository of well‐characterized participants with confirmed clinical distal, symmetrical polyneuropathy (DSP) as ascertained by peripheral nerve specialists at seven major academic centers in the United States (Icahn School of Medicine at Mount Sinai, Johns Hopkins University School of Medicine, Northwestern University, Kansas University Medical Center, University of Michigan, University of Utah, and Washington University School of Medicine) that allows for robust cross‐sectional observational data. Participants were identified at the discretion of the site principal investigator, often in the setting of the clinic or the electromyography lab. The registry only enrolls participants with type 1 and type 2 DPN, prediabetes, and CSPN. For the purposes of this study, we used the term ‘NDPN’ to refer to participants with prediabetes and CSPN. All participants completed a questionnaire regarding neuropathy symptoms, medical, family, and social history, as well as a MetS inventory. The questionnaire included detailed information about neuropathic symptoms such as pain quality and intensity, numbness, paresthesia, allodynia, weakness, balance, and dysautonomia. A neurologic examination was performed, which included testing of proprioception (normal, reduced, absent), vibration (Rydel‐Seiffer), and pinprick sensation (normal, reduced, absent) at the toes, ankles, and fingers. Additional assessments included motor strength testing (Medical Research Council), deep tendon reflexes, along with balance (Romberg) and gait examination (including tandem). The structured participant history questionnaire and physical examination form were developed by the PNRR group. After thoroughly analyzing the presence of neuropathic symptoms and signs, the neuromuscular expert determined whether the participant had peripheral neuropathy and if the participant was eligible for enrollment. A minimum laboratory data set for every subject was required, consisting of one glycemic test (hemoglobin A1c, fasting glucose, or 2‐h oral glucose tolerance test), B12 level, and serum protein electrophoresis with immunofixation. Lipid profile was recommended but not mandatory [21]. Nerve conduction studies (NCS) and skin biopsy evaluation of the distal leg site to evaluate intraepidermal nerve fiber density (IENFD) were performed, though not required. Participants with other confirmed causes of DSP, such as amyloidosis, chronic renal failure, alcohol abuse, vitamin deficiencies, or inherited neuropathies (based on genetic diagnosis or neuropathy in a first‐degree family member) were excluded, as were primary demyelinating neuropathies. Participants with a diagnosis of DPN or NDPN who were recruited between 2011 and 2023 from the seven consortia sites were included in our study.

### 2.2 Definition of MetS

The updated National Cholesterol Education Program/Adult Treatment Panel III criteria were used to define MetS and its individual components [22]. MetS was defined as the presence of at least 3 of the 5 MetS components: hyperglycemia, hypertension, elevated BMI (> 27), hypertriglyceridemia, and low high‐density lipoprotein (HDL) cholesterol. Hyperglycemia was confirmed by fasting plasma glucose (FPG) ≥ 100 mg/dL, Hemoglobin A1c (HbA1c) ≥ 5.7%, 2‐h Oral Glucose Tolerance Test (2 h GTT) ≥ 140 mg/dL, or prescription of anti‐hyperglycemic medications. Participants with hyperglycemia were further classified as diabetes (HbA1c ≥ 6.5%, FPG ≥ 126 mg/dL or 2 h GTT ≥ 200 mg/dL) or prediabetes (HbA1c ≥ 5.7%, FPG ≥ 100 or 2 h GTT ≥ 140 mg/dL). With regard to the other four MetS criteria: (1) hypertension was defined as a blood pressure ≥ 130/85 mmHg or prescription of an anti‐hypertensive agent; (2) elevated BMI (overweight) was defined by a body mass index (BMI) ≥ 27 (central obesity was not measured), as this cutoff has been shown to be ideal for the identification of MetS [23]; (3) hypertriglyceridemia was defined as a serum triglyceride level ≥ 150 mg/dL or the prescription of triglyceride‐lowering drugs (fibrates); and (4) low HDL cholesterol was defined as a serum HDL < 40 mg/dL in males and < 50 mg/dL in females or the prescription of a cholesterol‐lowering drug (statin). Participants who fulfilled the last two criteria by use of lipid‐lowering agents received a maximum of 1 point (even if taking both a fibrate and statin for high triglyceride and low HDL, respectively) [22, 24]. For the purposes of our study, we defined MetS burden as the number of MetS criteria fulfilled (0–5). When stratifying by glycemic status, we defined the MetS burden as the number of non‐glycemic MetS criteria fulfilled (0–4). For the analysis of MetS, we used an available case analysis approach, including only participants with complete lipid level data.

### 2.3 Outcomes

#### 2.3.1 Pain

Pain intensity was assessed using a 10‐point Numeric Rating Scale (NRS), where 0 represented no pain and 10 represented the most severe pain. Participants were considered to have pain if the NRS was greater than 0.

#### 2.3.2 Neuropathy Severity

We used the Total Neuropathy Score‐reduced (TNSr) to grade neuropathy severity. The TNSr has five components (symptom extension, pin sensibility, vibration sensibility, muscle strength, and tendon reflexes), and the score ranges from 0 (normal) to 20 (severe neuropathy). Participants were classified as having mild (1–8), moderate (9–15), or severe (16–20) neuropathy [25]. The TNSr carries an inter‐rater reliability of 0.80 [25].

#### 2.3.3 Fiber Type Classification

Fiber type was clinically defined as small fiber, large fiber, or mixed fiber based on neurological examination (signs), in accordance with the Analgesic, Anesthetic, and Addiction Clinical Trial Translations, Innovations, Opportunities and Networks (ACTTION) diagnostic criteria used for idiopathic DSP [26]. Small fiber was defined as the presence of decreased pinprick sensation with normal vibration and proprioception. Large fiber was defined as decreased vibration or proprioception with normal pinprick. Mixed fiber was defined as decreased pinprick sensation and decreased vibration or proprioception. Pinprick, vibration, and proprioception examination techniques and data collection methods are defined in the PNRR protocol [21].

### 2.4 Statistical Analysis

Descriptive statistics were used to summarize the demographic and clinical characteristics of the cohort, including metabolic profiles, stratified by neuropathy type (DPN or NDPN). Two‐sample *t*‐tests (for continuous variables) and Pearson's chi‐square tests (for categorical variables) were used to assess differences by neuropathy type.

### 2.5 Unadjusted Analysis

Pearson's chi‐square tests were used to determine the unadjusted differences in pain (yes/no), neuropathy severity (mild vs. moderate vs. severe), and the neuropathy fiber type (small vs. large vs. mixed) between those with DPN and NDPN. Similarly, Cochrane‐Armitage tests of trend were conducted to assess the unadjusted association between the trend of increasing MetS burden and presence of pain (yes/no), neuropathy severity (mild vs. non‐mild [moderate and severe]), and neuropathy fiber type (mixed vs. non‐mixed [small and large]) for the entire cohort, and stratified by glycemic group (normoglycemia, prediabetes, and diabetes).

### 2.6 Adjusted Analysis

Logistic regression models were fit to determine associations between the presence of pain and individual MetS components (glycemic status, triglycerides, HDL, systolic blood pressure [SBP], BMI) as well as between pain and non‐glycemic MetS burden (0–4), adjusted for glycemic status. Similarly, multiple linear regression models were fit to determine the associations between neuropathy severity and individual MetS components as well as between neuropathy severity and non‐glycemic MetS burden (0–4) adjusted for glycemic status. Lastly, multinomial logistic regression models were fit to determine the associations between fiber type (small fiber, large fiber, mixed fiber) and individual MetS components as well as between fiber type and the number of non‐glycemic MetS burdens (0–4) adjusted for glycemic status. Specifically, a single multinomial model was fit to separately determine differences in the odds of having large and mixed fiber neuropathy compared to small fiber neuropathy (SFN). All regression models were adjusted for age, sex, and height.

All analyses were performed in R version 4.2.2, and statistical significance for all tests was determined using two‐sided *p* values with a threshold of 0.05. Only complete observations without missing information on fiber type and individual MetS components were used.

### 2.7 Sensitivity Analysis

Sensitivity analyses were completed on participants with NCS‐ and/or IENFD‐confirmed peripheral neuropathy to assess comparability to the entire study cohort, as 86% had NCS and 33% had IENFD measures. Abnormal NCS were defined by the presence of at least one abnormal parameter (conduction velocity, sensory nerve action potential amplitude, or sensory peak onset latency for sensory nerves, or conduction velocity, distal motor onset latency, compound muscle action potential, or F‐latency for motor nerves) in two out of three of the following nerves: sural sensory, ulnar sensory, and/or peroneal motor nerves, respectively, using an approach codified by Dyck et al. [27] Site‐specific normative values (for each of the seven EMG labs) were used to determine abnormality for NCS [21]. Only IENFD data from the distal leg site was used to determine the presence or absence of small fiber involvement (normal/abnormal) using fifth percentile cutoffs [28]. In a sensitivity analysis for fiber type, among participants having both NCS and IENFD assessed, large‐fiber neuropathy was defined as abnormal NCS with normal IENFD, small‐fiber neuropathy as normal NCS with abnormal IENFD, and mixed‐fiber neuropathy as abnormal NCS and IENFD.

### 2.8 Standard Protocol Approvals, Registrations, and Participation Consents

PNRR is approved by each site's Institutional Review Boards. All participants provided written informed consent prior to enrollment.

## 3 Results

### 3.1 Study Participation, Demographic Information, and Missing Data

A total of 1112 participants were enrolled in this study. 265 (23.8%) had DPN and 847 (76.2%) had NDPN. Demographic characteristics are described in Table 1. The mean (standard deviation [SD]) age was 62.6 (13.2) years, and 60.6% were male. Participants with normoglycemia accounted for 47.8% of the population, prediabetes for 28.3%, and diabetes for 23.8%. MetS was present in 62.8% of all participants (Table 2). Missing data included 305 (27.4%) HDL, 300 (27%) triglycerides, 106 (9.5%) HbA1c, and 8 (0.7%) blood pressure measurements. Additionally, some participants had unknown race (3, 0.03%), ethnicity (4, 0.04%), and neuropathy duration (19, 1.7%). A total of 815 (73.3%) participants had complete data to determine each MetS component.

**TABLE 1:** Participant demographics stratified by diagnosis.

Variable	Full cohort (*N* = 1112)	Diabetic neuropathy (*N* = 265)	NDPN (*N* = 847)	*p*
Age, mean (SD)	62.6 (13.2)	61.8 (12.7)	62.8 (13.3)	0.25
Sex, *n* (%)				
Male	647 (60.6)	167 (63.0)	507 (59.9)	0.39
Female	438 (39.4)	98 (37.0)	340 (40.1)	
Hispanic, *n* (%)				
No	1088 (98.2)	252 (95.8)	836 (98.9)	< 0.01
Yes	20 (1.8)	11 (4.2)	9 (1.1)	
Race, *n* (%)				
American Indian/Alaska Native	3 (0.3)	1 (0.4)	2 (0.2)	< 0.01
Asian	14 (1.3)	3 (1.1)	11 (1.3)	
Black	67 (6.0)	39 (14.8)	28 (3.3)	
White	1013 (91.3)	215 (81.7)	798 (94.3)	
More than one race	12 (1.1)	5 (1.9)	7 (0.8)	

Abbreviations: NDPN, non‐diabetic peripheral neuropathy; SD, standard deviation.

**TABLE 2:** Metabolic syndrome components stratified by diagnosis.

Variable	Full cohort (*N* = 1112)	Diabetic neuropathy (*N* = 265)	NDPN (*N* = 847)	*p*
SBP, mean (SD)	131.6 (17.3)	134.7 (18.4)	130.7 (16.8)	< 0.01
DBP, mean (SD)	76.3 (10.6)	77.3 (10.9)	76.0 (10.5)	0.1
Triglycerides, mean (SD)	140.9 (96.8)	174.1 (113.9)	129.5 (87.4)	< 0.01
HDL, mean (SD)	52.7 (18.3)	46.5 (14.9)	54.9 (18.8)	< 0.01
BMI, mean (SD)	29.5 (6.4)	32.2 (6.7)	28.7 (6.1)	< 0.01
HbA1c, mean (SD)	6.0 (1.3)	7.3 (1.8)	5.5 (0.4)	< 0.01
Glycemic status, *n* (%)				
Diabetes	265 (23.8)	265 (100.0)	0 (0.0)	< 0.01
Normoglycemia	532 (47.8)	0 (0.0)	532 (62.8)	
Prediabetes	315 (28.3)	0 (0.0)	315 (37.2)	
Diabetes type, *n* (%)				
Type 1	20 (1.8)	20 (7.5)	0 (0.0)	< 0.01
Type 2	225 (20.2)	225 (84.9)	0 (0.0)	
Unspecified	20 (1.8)	20 (7.5)	0 (0.0)	
MetS prevalence, *n* (%)				
No	310 (38.0)	17 (8.2)	293 (48.2)	< 0.01
Yes	505 (62.8)	190 (91.8)	315 (51.8)	

Abbreviations: BMI, body mass index; DBP, diastolic blood pressure; HbA1c, Hemoglobin A1c; HDL, high‐density lipoprotein; MetS, metabolic syndrome; NDPN, non‐diabetic peripheral neuropathy; SBP, systolic blood pressure; SD, standard deviation.

### 3.2 Comparison of DPN and NDPN

Participants with DPN were more likely to be Hispanic (4.2% vs. 1.1%, *p* < 0.01), Black (14.8% vs. 3.3%, *p* < 0.01), and carry a higher MetS prevalence (91.8% vs. 51.8%, *p* < 0.01) in comparison to those with NDPN (Tables 1 and 2). DPN participants reported more allodynia, balance difficulty, abnormal gait, general weakness, as well as interossei and ADM weakness on exam (all *p* < 0.01), although no significant differences were seen in the prevalence of ankle dorsiflexion, great toe dorsiflexion, or great toe plantarflexion weakness (Table 3).

**TABLE 3:** Clinical findings associated with neuropathy stratified by diagnosis.

Variable	Full cohort (*N* = 1112)	Diabetic neuropathy (*N* = 265)	NDPN (*N* = 847)	*p*
TNSr, mean (SD)	6.9 (3.3)	7.3 (3.4)	6.6 (3.3)	< 0.01
Neuropathy duration, years, mean (SD)	6.29 (6.29)	6.92 (6.85)	6.09 (6.10)	0.06
Fiber type, *n* (%)				
Large	151 (13.6)	26 (9.8)	125 (14.8)	< 0.01
Mixed	657 (59.1)	184 (69.4)	473 (55.8)	
Small	162 (14.6)	32 (12.1)	130 (15.3)	
Unknown	142 (12.8)	23 (8.7)	119 (14.0)	
Pain—yes, *n* (%)	851 (76.5)	225 (84.9)	626 (73.9)	< 0.01
Pain intensity, mean (SD)	5.7 (2.5)	6.5 (2.2)	5.5 (2.5)	< 0.01
Allodynia (self‐report)—yes, *n* (%)	440 (51.8)	132 (58.9)	308 (49.3)	0.02
Numbness (self‐report)—yes, *n* (%)	991 (89.1)	238 (89.8)	753 (88.9)	0.76
Balance difficulties (self‐report)—yes, *n* (%)	681 (61.4)	185 (69.8)	496 (58.7)	< 0.01
Weakness (self‐report)—yes, *n* (%)	566 (51.0)	156 (58.9)	410 (48.5)	< 0.01
Gait (exam)—impaired, *n* (%)	236 (21.2)	73 (27.5)	163 (19.2)	< 0.01
Ankle dorsiflexion (exam)—weak, *n* (%)	118 (10.6)	31 (11.7)	87 (10.3)	0.59
Great toe dorsiflexion (exam)—weak, *n* (%)	230 (20.8)	64 (24.4)	166 (19.6)	0.11
Finger abduction (exam)—weak, *n* (%)	88 (7.9)	34 (12.8)	54 (6.4)	< 0.01

Abbreviations: NDPN, non‐diabetic peripheral neuropathy; SD, standard deviation; TNSr, total neuropathy score reduced.

#### 3.2.1 Pain

Participants with DPN reported more pain (84.9% vs. 73.9%, *p* < 0.01) and a higher mean pain intensity (6.5 [2.2] vs. 5.5 [2.5], *p* < 0.01) compared to those with NDPN (Table 3). Logistic regression revealed that participants with diabetes had increased adjusted odds of pain compared to participants with normoglycemia (odds ratio [OR] 1.85, 95% CI: 1.15–3.03) (Table S1). This association did not meaningfully change after adjusting for neuropathy severity (OR 1.86, 95% CI: 1.16–3.06).

#### 3.2.2 Neuropathy Severity

Participants with DPN had a higher neuropathy severity score (TNSr) as compared to those with NDPN (7.3 [3.4] vs. 6.6 [3.3], *p* < 0.01) (Table 3). Linear regression analysis also showed an increase in the TNSr in participants with diabetes compared to those with normoglycemia (point estimate [PE] 0.84, 95% CI: 0.27–1.42), after adjusting for triglycerides, HDL, SBP, and BMI (Table S2).

#### 3.2.3 Fiber Type

Participants with DPN had more mixed fiber involvement as compared with NDPN participants (69.4% vs. 55.8%, *p* < 0.01). Multinomial logistic regression showed that participants with diabetes had increased odds of having mixed fiber versus small fiber involvement (OR 1.82, 95% CI: 0.99–3.36) compared to participants with normoglycemia, although the finding was not statistically significant (Table S3).

### 3.3 Impact of MetS Burden and Individual MetS Components on Neuropathy Outcomes

#### 3.3.1 Pain

**FIGURE 1:**
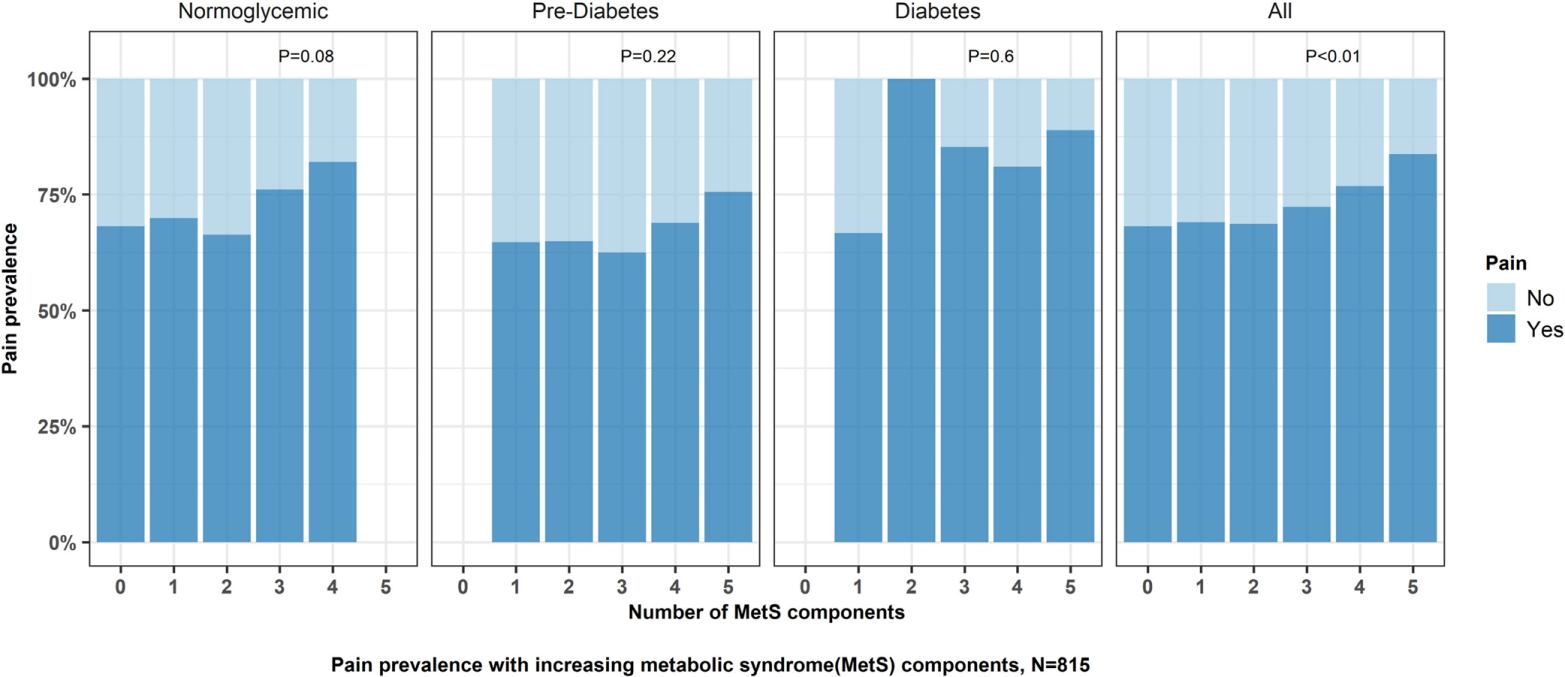
Prevalence of pain with increasing metabolic syndrome (MetS) components. Includes only complete observations with pain prevalence and MetS score. Overall *N* = 815, individual sample size based on glycemic status, −Normoglycemia (*N* = 360), Prediabetes (*N* = 248), Diabetes (*N* = 207). *p* values based on the chi‐square test for differences in pain prevalence with increasing MetS components within each group.

As MetS burden increased, the prevalence of pain increased (*p* < 0.01 for trend, Figure 1). Logistic regression revealed there was a significant association between increasing non‐glycemic MetS burden (0–4) and odds of having pain (OR 1.23, 95% CI: 1.06–1.41) (Table S1).

#### 3.3.2 Neuropathy Severity

**FIGURE 2:**
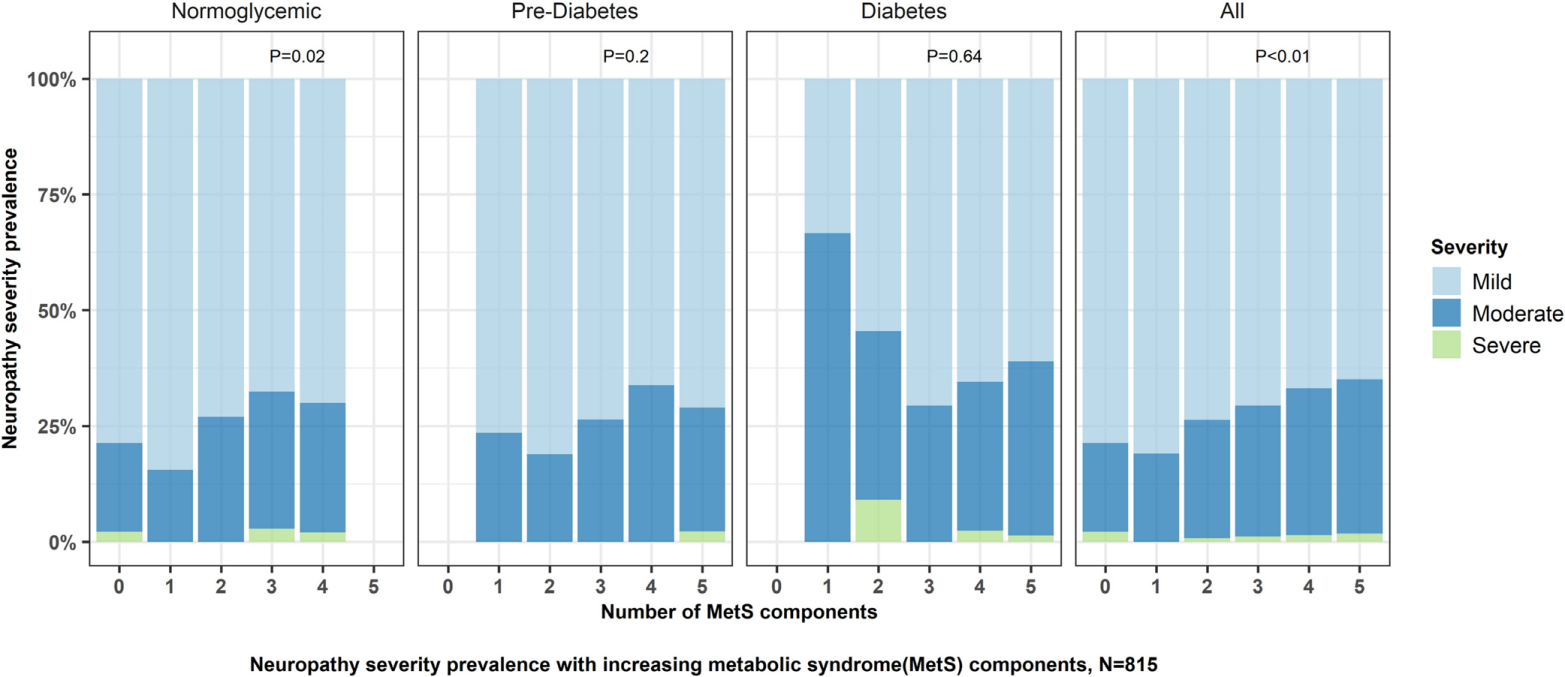
Prevalence of metabolic syndrome (MetS) components with increasing neuropathy severity. Severity is characterized by total neuropathy severity score (reduced), Mild = 0–8, Moderate = 9–15, severe = 16–20. Includes only complete observations with TNSr and MetS scores. Overall *N* = 815, individual sample size based on glycemic status, normoglycemia (*N* = 360), prediabetes (*N* = 248), diabetes (*N* = 207). *p* values based on the chi‐square test for differences in the number of MetS components with increasing neuropathy severity within each group.

As MetS burden increased, the severity of neuropathy increased (*p* < 0.01 for trend, Figure 2). When stratified by glycemic status, the neuropathy severity increased with increasing MetS burden in participants with normoglycemia (*p* = 0.02), but not in those with pre‐diabetes or diabetes. Linear regression did not reveal an association between increasing non‐glycemic MetS burden and neuropathy severity (PE 0.17, 95% CI: −0.02 to 0.36). Besides diabetes, increasing BMI was also associated with worse neuropathy severity (PE 0.05, 95% CI: 0.02 to 0.09) (Table S2).

#### 3.3.3 Fiber Type

**FIGURE 3:**
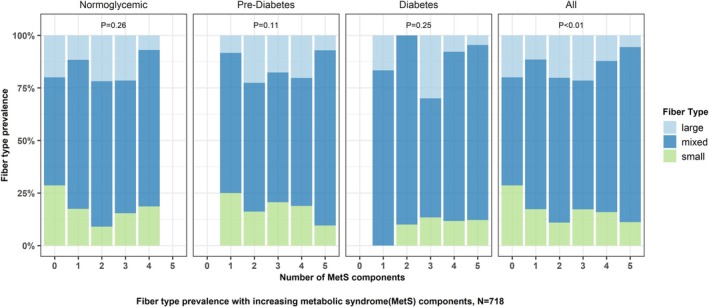
Prevalence of fiber type with increasing metabolic syndrome (MetS) components. Includes only complete observations with fiber type and MetS score. Overall sample size = 718, individual sample size based on glycemic status, normoglycemia (*N* = 307), prediabetes (*N* = 222), diabetes (*N* = 189). *p* values based on the chi‐square test for differences in fiber type prevalence with increasing MetS components within each group.

As the MetS burden increased, the prevalence of mixed fiber involvement increased (*p* < 0.01 for trend, Figure 3). Multinomial logistic regression analysis did not reveal a significant association between increasing non‐glycemic MetS burden (0–4) and odds of having mixed fiber (OR 1.1, 95% CI: 0.91–1.32) involvement. The multinomial regression model with individual MetS components indicated that participants with prediabetes were more likely to have SFN than mixed fiber, compared to those with normoglycemia (OR 0.59, 95% CI: 0.35–0.99) (Table S3).

### 3.4 Sensitivity Analysis

A total of 727 participants with confirmed peripheral neuropathy were included in the pain and neuropathy severity sensitivity analysis. Additionally, 254 participants with both NCS and IENFD data were included in the fiber‐type sensitivity analysis.

#### 3.4.1 Pain

Similar to the primary analysis, logistic regression analysis showed that persons with diabetes had higher odds of experiencing pain compared to persons with normoglycemia (OR 1.43, 95% CI: 0.82–2.55), though this finding was no longer statistically significant. Additionally, a significant association between an increasing non‐glycemic MetS burden (0–4) and the odds of experiencing pain (OR 1.43, 95% CI: 1.19–1.73) was observed.

#### 3.4.2 Neuropathy Severity

Similar to the primary analysis, linear regression analysis showed that persons with diabetes (PE 0.66, 95% CI: −0.08 to 1.41) and those with an increasing non‐glycemic MetS burden (PE 0.13, 95% CI: −0.13 to 0.38) had higher neuropathy severity, though these findings were no longer statistically significant.

#### 3.4.3 Fiber Type

Multinomial logistic regression showed that persons with diabetes had increased odds of having large fiber versus small fiber involvement (OR 8.43, 95% CI: 1.01–70.6) and mixed fiber versus small fiber involvement (OR 10.96, 95% CI: 3.12–38.49) compared to participants with normoglycemia. However, multinomial logistic regression analysis did not show a significant association between increasing non‐glycemic MetS burden (0–4) and the odds of having large fiber (OR 1.13, 95% CI: 0.67–1.89) or mixed fiber involvement (OR 0.89, 95% CI: 0.64–1.24).

## 4 Discussion

We found that increasing MetS burden leads to more painful and possibly more severe neuropathy, with greater mixed fiber involvement. Similarly, diabetes leads to a more painful and severe neuropathy compared to NDPN and possibly to more mixed fiber involvement. Importantly, our sensitivity analyses revealed comparable findings. No prior studies have evaluated the effects of MetS burden on pain, neuropathy severity, and fiber type, or compared it to diabetes in patients with established polyneuropathy. Our findings highlight the similarities between DPN and the neuropathy associated with increasing MetS burden, which carry important implications for the recognition and treatment of these two conditions.

Our study showed that diabetes and increasing non‐glycemic MetS burden confer an increased likelihood of developing pain. The associations between pain, DPN, and NDPN have been explored in two smaller cross‐sectional studies from Canada and Denmark [29, 30]. The Canadian study also found a higher prevalence of pain in DPN [29], while the Danish study did not show a difference [30]. This discrepancy could be related to the different study populations, as DPN pain prevalence in Denmark is lower than in the USA [7, 31]. Other studies have explored pain prevalence in DPN cohorts without an NDPN comparator. The cross‐sectional Pain in Neuropathy Study (PiNS) found that moderately to severely painful DPN had higher HbA1c levels and that individuals were of a younger age, as compared to those with mildly painful or nonpainful DPN [32]. An Italian study showed that longer diabetes disease duration and higher HbA1c contributed to painful DPN, but this was also true of nonpainful DPN [33]. Even though no studies specifically focused on MetS burden (as an aggregate) and pain, some have explored the relationship between individual MetS components and pain. The Utah Diabetic Neuropathy Study (UDNS) and two European studies showed that BMI directly correlated with pain [24, 33, 34], although the PiNS study did not find such an association [32]. Furthermore, the Belgian cohort showed that low HDL cholesterol and high triglyceride levels are associated with painful DPN [34]. While we did not find an association between pain and individual non‐glycemic MetS components, our findings suggest that the aggregate effect of the number of MetS criteria fulfilled—what we refer to in our paper as MetS burden—is an independent risk factor for pain, alongside diabetes.

We found that neuropathy severity was driven by diabetes, overweight status (measured by BMI), and possibly by increasing non‐glycemic MetS burden. Two prior studies have briefly compared neuropathy severity between DPN and NDPN using the Utah Early Neuropathy Scale (UENS). No studies, however, have assessed the association between neuropathy severity, MetS burden, or individual MetS components. Regarding DPN and NDPN, the Canadian study did not show a difference in severity at baseline, but individuals with DPN developed a more severe neuropathy after 3 years [29]. The Danish cohort showed a higher UENS score in DPN individuals, but it was not statistically significant after adjusting for sex and age [30]. The UDNS only evaluated individuals with diabetes and found that neuropathy severity, measured by the UENS, correlated with diabetes duration and HbA1c levels [24]. Most of these results are consistent with our findings, suggesting that diabetes is an independent driver of neuropathy severity. Many studies have firmly established obesity and MetS burden as a risk factor for polyneuropathy prevalence [10, 13, 24, 35]. However, only the UDNS has explored MetS components and neuropathy severity, showing an association between weight and severity in individuals with diabetes [24]. Our study suggests that obesity and possibly MetS burden are associated with neuropathy severity, independent of glycemic status.

With regards to fiber type, diabetes and increasing MetS burden possibly led to more mixed fiber involvement, although adjusted analyses did not confirm this association. The UDNS showed that higher BMI and triglycerides correlated with small fiber neuropathy outcomes (analyzed by intraepidermal nerve fiber density [IENFD]), but they did not categorize individuals into small, large, or mixed fiber neuropathy [24]. In our cohort, unadjusted analysis did suggest that higher MetS burden and diabetes contributed to more of a mixed neuropathy phenotype, but future studies are needed to confirm this relationship.

DPN and MetS neuropathy appear to have a similar phenotype, causing a more painful and severe neuropathy, with more predisposition to mixed‐fiber involvement in comparison to NDPN. MetS burden plays an important role in the pathogenesis of DPN in type 2 diabetes [11, 12, 24], therefore, it is not surprising that DPN and MetS neuropathy are clinically similar. This may have important therapeutic implications. In individuals with MetS risk factors with or without diabetes, treatment should likely focus on disease‐modifying interventions that target all MetS components and not just hyperglycemia. While clinicians are aware that diabetes causes neuropathy, they should also look for metabolic neuropathy in those without diabetes.

Our study faced certain limitations. The cross‐sectional study design did not allow us to evaluate neuropathy progression. Generalizability is limited to individuals attending academic neurology centers and consenting to participation in the registry. Furthermore, we only included one glucose measurement (FPG, HbA1c, or 2 h GTT) to define prediabetes rather than confirming the diagnosis with a second glucose measurement, which was a limitation. Data on exercise level and the number of pain medications used were unavailable for many patients, limiting our ability to adjust for these two potential confounders. Finally, we did not have data to measure central obesity, and around a quarter of individuals lacked information on HDL and triglyceride levels. The relatively small number of participants with type 1 diabetes compared to those with type 2 diabetes also precluded a formal comparison between these two subgroups. Despite these limitations, it is worth noting that our DPN and NDPN cohorts had comparable durations of neuropathy symptoms, further highlighting the centrality of MetS burden as a key driver of neuropathy severity, alongside hyperglycemia.

In conclusion, increasing MetS burden leads to more painful neuropathy and possibly more severe neuropathy with more mixed fiber involvement. In addition, diabetes leads to a more painful and severe neuropathy compared to NDPN, and possibly more mixed fiber involvement. DPN and MetS neuropathy appear to have a similar phenotype, different from NDPN, which emphasizes the need for a multimodal therapeutic approach targeting all MetS risk factors for the prevention and treatment of these neuropathies.

## Author Contributions

**Long Davalos:** conceptualization, methodology, investigation, writing – original draft, visualization, project administration. **Brian C. Callaghan:** conceptualization, methodology, formal analysis, writing – review and editing, visualization, supervision. **Lavanya Muthukumar:** methodology, software, formal analysis, writing – original draft. **Simone Thomas:** conceptualization, investigation, resources, writing – review and editing, project administration. **Evan L. Reynolds:** methodology, software, formal analysis, writing – review and editing. **A. Gordon Smith:** conceptualization, methodology, investigation, writing – review and editing. **J. Robinson Singleton:** conceptualization, methodology, investigation, writing – review and editing. **Ahmet Höke:** conceptualization, methodology, investigation, writing – review and editing. **Senda Ajroud‐Driss:** investigation, writing – review and editing. **Mazen M. Dimachkie:** investigation, writing – review and editing. **Stefanie Geisler:** investigation, writing – review and editing. **David M. Simpson:** investigation, writing – review and editing. **Amro M. Stino:** conceptualization, methodology, investigation, writing – original draft, writing – review and editing, visualization, supervision.

## Conflicts of Interest

B.C.C. consults for DynaMed, performs medical legal consultations, including consultations for the Vaccine Injury Compensation Program, and receives research and editorial support from the American Academy of Neurology. A.G.S. reported receiving consulting fees from Merz, Sangamo, Argenx, and Alexion and data monitoring board fees from Eidos and Lexicon. A.H. consults for Pfizer, GenEdit, Eikonizo, HDAX Therapeutics, and Sangamo and receives editorial support from the American Neurological Association and research funding from DOD, NINDS, Dr. Miriam and Sheldon G. Adelson Medical Research Foundation, Merkin Family Foundation, and Foundation for Peripheral Neuropathy. S.A.‐D. served on the advisory board for Amylyx Pharmaceutical and Orphazyme, served as a speaker for Biogen, and received honoraria from MDA, AANEM, and Medscape. M.M.D. consults for Abata/Third Rock, Abcuro, Amicus, ArgenX, Astellas, Cabaletta Bio, Catalyst, CNSA, Covance/Labcorp, CSL‐Behring, Dianthus, Horizon, EMD Serono/Merck, Ig Society Inc., Ipsen, Janssen, Medlink, Nuvig, Octapharma, Priovant, Sanofi Genzyme, Shire Takeda, TACT/Treat NMD, UCB Biopharma, Valenza Bio, and Wolters Kluwer Health/UpToDate and has received research grants or contracts, or educational grants from Alexion/AstraZeneca, Alnylam Pharmaceuticals, Amicus, Argenx, Bristol‐Myers Squibb, Catalyst, CSL‐Behring, FDA/OOPD, GlaxoSmithKline, Genentech, Grifols, Mitsubishi Tanabe Pharma, MDA, NIH, Novartis, Octapharma, Orphazyme, Ra Pharma/UCB, Sanofi Genzyme, Sarepta Therapeutics, Shire Takeda, Spark Therapeutics, The Myositis Association, and UCB Biopharma/RaPharma. A.M.S. has consulted for Argenx, CSL Behring, Takeda, Sanofi, Immunovant, Annexon, and has received research support from Bristol Myers Squibb and the GBS‐CIDP Foundation. L.D., L.M., S.T., E.L.R., J.R.S., S.G., and D.M.S. have no relevant conflicts of interest to disclose.

## Supporting information


**Table S1.** Logistic regression for association between pain prevalence and metabolic syndrome components, adjusting for age, sex, and height.

**Table S2.** Linear regression for association between neuropathy severity and metabolic syndrome components, adjusting for age, sex, and height.

**Table S3.** Multinomial Logistic regression for association between Fiber type and metabolic syndrome components, adjusting for age, sex, and height.

## Data Availability

The data that support the findings of this study are available from the Peripheral Neuropathy Research Registry (PNRR). Restrictions apply to the availability of these data, which were used under license for this study. Data are available from the authors at the URL with the permission of the PNRR and the Foundation for Peripheral Neuropathy.

## References

[1] R. Hanewinckel , M. van Oijen , M. A. Ikram , and P. A. van Doorn , “The Epidemiology and Risk Factors of Chronic Polyneuropathy,” European Journal of Epidemiology 31, no. 1 (2016): 5–20. 26700499 10.1007/s10654-015-0094-6 PMC4756033

[2] J. Partanen , L. Niskanen , J. Lehtinen , E. Mervaala , O. Siitonen , and M. Uusitupa , “Natural History of Peripheral Neuropathy in Patients With Non‐Insulin‐Dependent Diabetes Mellitus,” New England Journal of Medicine 333, no. 2 (1995): 89–94. 7777034 10.1056/NEJM199507133330203

[3] G. M. Franklin , L. B. Kahn , J. Baxter , et al., “Sensory Neuropathy in Non‐Insulin‐Dependent Diabetes Mellitus. The San Luis Valley Diabetes Study,” American Journal of Epidemiology 131, no. 4 (1990): 633–643. 2316495 10.1093/oxfordjournals.aje.a115547

[4] P. J. Dyck , K. M. Kratz , J. L. Karnes , et al., “The Prevalence by Staged Severity of Various Types of Diabetic Neuropathy, Retinopathy, and Nephropathy in a Population‐Based Cohort: The Rochester Diabetic Neuropathy Study,” Neurology 43, no. 4 (1993): 817–824. 8469345 10.1212/wnl.43.4.817

[5] GBD 2021 Nervous System Disorders Collaborators , “Global, Regional, and National Burden of Disorders Affecting the Nervous System, 1990–2021: A Systematic Analysis for the Global Burden of Disease Study 2021,” Lancet Neurology 23, no. 4 (2024): 344–381. 38493795 10.1016/S1474-4422(24)00038-3 PMC10949203

[6] International Diabetes Federation , IDF Diabetes Atlas, 10th ed. (International Diabetes Federation, 2021).

[7] M. Kiyani , Z. Yang , L. T. Charalambous , et al., “Painful Diabetic Peripheral Neuropathy: Health Care Costs and Complications From 2010 to 2015,” Neurology Clinical Practice 10, no. 1 (2020): 47–57. 32190420 10.1212/CPJ.0000000000000671 PMC7057074

[8] M. Kazamel , A. M. Stino , and A. G. Smith , “Metabolic Syndrome and Peripheral Neuropathy,” Muscle & Nerve 63, no. 3 (2021): 285–293. 33098165 10.1002/mus.27086

[9] M. Pasnoor , M. M. Dimachkie , and R. J. Barohn , “Cryptogenic Sensory Polyneuropathy,” Neurologic Clinics 31, no. 2 (2013): 463–476. 23642719 10.1016/j.ncl.2013.01.008 PMC4090929

[10] B. C. Callaghan , L. Gao , Y. Li , et al., “Diabetes and Obesity Are the Main Metabolic Drivers of Peripheral Neuropathy,” Annals of Clinical Translational Neurology 5, no. 4 (2018): 397–405. 29687018 10.1002/acn3.531 PMC5899909

[11] B. C. Callaghan , R. Xia , E. Reynolds , et al., “Association Between Metabolic Syndrome Components and Polyneuropathy in an Obese Population,” JAMA Neurology 73, no. 12 (2016): 1468–1476. 27802497 10.1001/jamaneurol.2016.3745 PMC5217829

[12] B. C. Callaghan , R. Xia , M. Banerjee , et al., “Metabolic Syndrome Components Are Associated With Symptomatic Polyneuropathy Independent of Glycemic Status,” Diabetes Care 39, no. 5 (2016): 801–807. 26965720 10.2337/dc16-0081 PMC4839175

[13] R. Hanewinckel , J. Drenthen , S. Ligthart , et al., “Metabolic Syndrome Is Related to Polyneuropathy and Impaired Peripheral Nerve Function: A Prospective Population‐Based Cohort Study,” Journal of Neurology, Neurosurgery, and Psychiatry 87, no. 12 (2016): 1336–1342. 27656045 10.1136/jnnp-2016-314171

[14] B. Lu , J. Hu , J. Wen , et al., “Determination of Peripheral Neuropathy Prevalence and Associated Factors in Chinese Subjects With Diabetes and Pre‐Diabetes—ShangHai Diabetic neuRopathy Epidemiology and Molecular Genetics Study (SH‐DREAMS),” PLoS One 8, no. 4 (2013): e61053. 23613782 10.1371/journal.pone.0061053 PMC3628856

[15] S. Schlesinger , C. Herder , J. M. Kannenberg , et al., “General and Abdominal Obesity and Incident Distal Sensorimotor Polyneuropathy: Insights Into Inflammatory Biomarkers as Potential Mediators in the KORA F4/FF4 Cohort,” Diabetes Care 42, no. 2 (2019): 240–247. 30523031 10.2337/dc18-1842

[16] D. H. Christensen , S. T. Knudsen , S. S. Gylfadottir , et al., “Metabolic Factors, Lifestyle Habits, and Possible Polyneuropathy in Early Type 2 Diabetes: A Nationwide Study of 5,249 Patients in the Danish Centre for Strategic Research in Type 2 Diabetes (DD2) Cohort,” Diabetes Care 43, no. 6 (2020): 1266–1275. 32295810 10.2337/dc19-2277

[17] N. A. Visser , A. F. J. E. Vrancken , Y. T. van der Schouw , L. H. van den Berg , and N. C. Notermans , “Chronic Idiopathic Axonal Polyneuropathy Is Associated With the Metabolic Syndrome,” Diabetes Care 36, no. 4 (2013): 817–822. 23204246 10.2337/dc12-0469 PMC3609524

[18] B. C. Callaghan , E. Reynolds , M. Banerjee , E. Chant , E. Villegas‐Umana , and E. L. Feldman , “Central Obesity Is Associated With Neuropathy in the Severely Obese,” Mayo Clinic Proceedings 95, no. 7 (2020): 1342–1353. 32622444 10.1016/j.mayocp.2020.03.025 PMC7340115

[19] E. L. Reynolds , B. C. Callaghan , M. Banerjee , E. L. Feldman , and V. Viswanathan , “The Metabolic Drivers of Neuropathy in India,” Journal of Diabetes and Its Complications 34, no. 10 (2020): 107653. 32624332 10.1016/j.jdiacomp.2020.107653 PMC7502489

[20] E. L. Reynolds , G. Akinci , M. Banerjee , et al., “The Determinants of Complication Trajectories in American Indians With Type 2 Diabetes,” JCI Insight 6, no. 10 (2021): e146849. 34027894 10.1172/jci.insight.146849 PMC8262294

[21] S. Thomas , S. Ajroud‐Driss , M. M. Dimachkie , et al., “Peripheral Neuropathy Research Registry: A Prospective Cohort,” Journal of the Peripheral Nervous System 24, no. 1 (2019): 39–47. 30629307 10.1111/jns.12301

[22] R. J. Lipsy , “The National Cholesterol Education Program Adult Treatment Panel III Guidelines,” Journal of Managed Care Pharmacy 9, no. 1 Suppl (2003): 2–5. 10.18553/jmcp.2003.9.s1.2 PMC10437161 14613351

[23] O. Kobo , R. Leiba , O. Avizohar , and A. Karban , “Normal Body Mass Index (BMI) Can Rule Out Metabolic Syndrome: An Israeli Cohort Study,” Medicine (Baltimore) 98, no. 9 (2019): e14712. 30817613 10.1097/MD.0000000000014712 PMC6831180

[24] A. G. Smith and J. R. Singleton , “Obesity and Hyperlipidemia Are Risk Factors for Early Diabetic Neuropathy,” Journal of Diabetes and Its Complications 27, no. 5 (2013): 436–442. 23731827 10.1016/j.jdiacomp.2013.04.003 PMC3766404

[25] E. M. L. Smith , J. A. Cohen , M. A. Pett , and S. L. Beck , “The Reliability and Validity of a Modified Total Neuropathy Score‐Reduced and Neuropathic Pain Severity Items When Used to Measure Chemotherapy‐Induced Peripheral Neuropathy in Patients Receiving Taxanes and Platinums,” Cancer Nursing 33, no. 3 (2010): 173–183. 20357656 10.1097/NCC.0b013e3181c989a3

[26] R. Freeman , J. S. Gewandter , C. G. Faber , et al., “Idiopathic Distal Sensory Polyneuropathy,” Neurology 95, no. 22 (2020): 1005–1014. 33055271 10.1212/WNL.0000000000010988 PMC7734920

[27] P. J. Dyck , R. E. Carter , and W. J. Litchy , “Modeling Nerve Conduction Criteria for Diagnosis of Diabetic Polyneuropathy,” Muscle & Nerve 44, no. 3 (2011): 340–345. 21996793 10.1002/mus.22074 PMC3193597

[28] G. Lauria , M. Bakkers , C. Schmitz , et al., “Intraepidermal Nerve Fiber Density at the Distal Leg: A Worldwide Normative Reference Study,” Journal of the Peripheral Nervous System 15, no. 3 (2010): 202–207. 21040142 10.1111/j.1529-8027.2010.00271.x

[29] S. Sachedina and C. Toth , “Progression in Idiopathic, Diabetic, Paraproteinemic, Alcoholic, and B12 Deficiency Neuropathy,” Journal of the Peripheral Nervous System 18, no. 3 (2013): 247–255. 24028193 10.1111/jns5.12042

[30] M. Itani , S. Gylfadottir , T. Krøigård , et al., “Comparison of Diabetic and Idiopathic Sensory Polyneuropathies With Respect to Nerve Fibre Affection and Risk Factors,” BMJ Neurology Open 4, no. 1 (2022): e000247. 10.1136/bmjno-2021-000247 PMC8921860 35360409

[31] S. S. Gylfadottir , M. Itani , T. Krøigård , et al., “Diagnosis and Prevalence of Diabetic Polyneuropathy: A Cross‐Sectional Study of Danish Patients With Type 2 Diabetes,” European Journal of Neurology 27, no. 12 (2020): 2575–2585. 32909392 10.1111/ene.14469

[32] A. C. Themistocleous , J. D. Ramirez , P. R. Shillo , et al., “The Pain in Neuropathy Study (PiNS): A Cross‐Sectional Observational Study Determining the Somatosensory Phenotype of Painful and Painless Diabetic Neuropathy,” Pain 157, no. 5 (2016): 1132–1145. 27088890 10.1097/j.pain.0000000000000491 PMC4834814

[33] A. Truini , V. Spallone , R. Morganti , et al., “A Cross‐Sectional Study Investigating Frequency and Features of Definitely Diagnosed Diabetic Painful Polyneuropathy,” Pain 159, no. 12 (2018): 2658–2666. 30161042 10.1097/j.pain.0000000000001378

[34] K. Van Acker , D. Bouhassira , D. De Bacquer , et al., “Prevalence and Impact on Quality of Life of Peripheral Neuropathy With or Without Neuropathic Pain in Type 1 and Type 2 Diabetic Patients Attending Hospital Outpatients Clinics,” Diabetes & Metabolism 35, no. 3 (2009): 206–213. 19297223 10.1016/j.diabet.2008.11.004

[35] T. T. W. van Herpt , A. Dehghan , M. van Hoek , et al., “The Clinical Value of Metabolic Syndrome and Risks of Cardiometabolic Events and Mortality in the Elderly: The Rotterdam Study,” Cardiovascular Diabetology 15 (2016): 69. 27117940 10.1186/s12933-016-0387-4 PMC4847340

