# Supplementary material for: The Impact of Diabetes and Metabolic Syndrome Burden on Pain, Neuropathy Severity and Fiber Type
Source: Ann Clin Transl Neurol. 2025 May 19;12(7):1408–17. doi: 10.1002/acn3.70072 (PMC12257117; doi:10.1002/acn3.70072)
Supplement: Supplementary file 1 — Table S1. Logistic regression for association between pain prevalence and metabolic syndrome components, adjusting for age, sex, and height. [file ACN3-12-1408-s003.docx]

**Table S1.** Logistic regression for association between pain prevalence and metabolic syndrome components, adjusting for age, sex, and height.

| Variable | N | OR | LCI | UCI |
| --- | --- | --- | --- | --- |
|  |  |  |  |  |
| Age | 799 | 0.96 | 0.95 | 0.98 |
| Male  (Ref: female) | 799 | 0.85 | 0.50 | 1.46 |
| Height (cm) | 799 | 0.98 | 0.95 | 1.00 |
| Diabetes  (Ref: Normal) | 799 | 1.85 | 1.15 | 3.03 |
| Prediabetes  (Ref: Normal) | 799 | 0.86 | 0.59 | 1.25 |
| Triglycerides (mg/dL) | 799 | 1.00 | 1.00 | 1.00 |
| HDL (mg/dL) | 799 | 0.99 | 0.98 | 1.00 |
| SBP (mm Hg) | 799 | 1.00 | 0.99 | 1.01 |
| BMI | 799 | 1.02 | 0.99 | 1.05 |

*N includes only complete observations without missing values in pain prevalence, and individual metabolic syndrome components.

Abbreviations: BMI, body mass index; HDL, high-density lipoprotein; SBP, systolic blood pressure.
