# Supplementary material for: The Impact of Diabetes and Metabolic Syndrome Burden on Pain, Neuropathy Severity and Fiber Type
Source: Ann Clin Transl Neurol. 2025 May 19;12(7):1408–17. doi: 10.1002/acn3.70072 (PMC12257117; doi:10.1002/acn3.70072)
Supplement: Supplementary file 2 — Table S2. Linear regression for association between neuropathy severity and metabolic syndrome components, adjusting for age, sex, and height. [file ACN3-12-1408-s001.docx]

**Table S2.** Linear regression for association between neuropathy severity and metabolic syndrome components, adjusting for age, sex, and height.

| Variable | N | Estimate | LCI | UCI |
| --- | --- | --- | --- | --- |
|  |  |  |  |  |
| Age | 799 | 0.05 | 0.03 | 0.07 |
| Male  (Ref: female) | 799 | 0.40 | -0.31 | 1.11 |
| Height (cm) | 799 | 0.03 | 0.00 | 0.06 |
| Diabetes  (Ref: Normal) | 799 | 0.84 | 0.27 | 1.42 |
| Prediabetes  (Ref: Normal) | 799 | -0.07 | -0.59 | 0.46 |
| Triglycerides (mg/dL) | 799 | 0.00 | 0.00 | 0.00 |
| HDL (mg/dL) | 799 | 0.01 | 0.00 | 0.02 |
| SBP (mm Hg) | 799 | 0.01 | 0.00 | 0.02 |
| BMI | 799 | 0.05 | 0.02 | 0.09 |

*N includes only complete observations without missing values in total neuropathy score, and individual metabolic syndrome components.

Abbreviations: BMI, body mass index; HDL, high-density lipoprotein; SBP, systolic blood pressure.
