# Supplementary material for: The Impact of Diabetes and Metabolic Syndrome Burden on Pain, Neuropathy Severity and Fiber Type
Source: Ann Clin Transl Neurol. 2025 May 19;12(7):1408–17. doi: 10.1002/acn3.70072 (PMC12257117; doi:10.1002/acn3.70072)
Supplement: Supplementary file 3 — Table S3. Multinomial Logistic regression for association between Fiber type and metabolic syndrome components, adjusting for age, sex, and height. [file ACN3-12-1408-s002.docx]

**Table S3.** Multinomial Logistic regression for association between Fiber type and metabolic syndrome components, adjusting for age, sex, and height.

|  | Large fiber vs small fiber | | | | Mixed fiber vs small fiber | | | |
| --- | --- | --- | --- | --- | --- | --- | --- | --- |
| Variable | N | OR | LCI | UCI | N | OR | LCI | UCI |
|  |  |  |  |  |  |  |  |  |
| Age | 705 | 1.09 | 1.06 | 1.11 | 705 | 1.07 | 1.05 | 1.09 |
| Male  (Ref: female) | 705 | 0.64 | 0.30 | 1.34 | 705 | 0.50 | 0.27 | 0.93 |
| Height (cm) | 705 | 1.05 | 1.03 | 1.07 | 705 | 1.07 | 1.05 | 1.09 |
| Diabetes  (Ref: Normal) | 705 | 1.37 | 0.61 | 3.05 | 705 | 1.82 | 0.99 | 3.36 |
| Prediabetes  (Ref: Normal) | 705 | 0.68 | 0.35 | 1.30 | 705 | 0.59 | 0.35 | 0.99 |
| Triglycerides (mg/dL) | 705 | 1.00 | 0.99 | 1.00 | 705 | 1.00 | 1.00 | 1.01 |
| Hdl (mg/dL) | 705 | 0.99 | 0.98 | 1.01 | 705 | 0.99 | 0.98 | 1.01 |
| SBP (mm Hg) | 705 | 1.00 | 0.99 | 1.02 | 705 | 1.01 | 0.99 | 1.02 |
| BMI | 705 | 0.96 | 0.91 | 1.01 | 705 | 1.00 | 0.96 | 1.03 |

*N includes only complete observations without missing values in fiber type, and individual MetS components.

Abbreviations: BMI, body mass index; HDL, high-density lipoprotein; SBP, systolic blood pressure.
